# Integrin-Specific Mechanoresponses to Compression and Extension Probed by Cylindrical Flat-Ended AFM Tips in Lung Cells

**DOI:** 10.1371/journal.pone.0032261

**Published:** 2012-02-23

**Authors:** Irene Acerbi, Tomás Luque, Alícia Giménez, Marta Puig, Noemi Reguart, Ramon Farré, Daniel Navajas, Jordi Alcaraz

**Affiliations:** 1 Unitat de Biofísica i Bioenginyeria, Facultat de Medicina, Universitat de Barcelona, Barcelona, Spain; 2 Laboratorio di Tecnologie Biomediche, Dipartimento di Bioingegneria, Politecnico di Milano, Milano, Italy; 3 Institut de Bioenginyeria de Catalunya (IBEC), Barcelona, Spain; 4 Institut d'Investigacions Biomèdiques August Pi i Sunyer (IDIBAPS), Barcelona, Spain; 5 CIBER de Enfermedades Respiratorias (CIBERES), Bunyola, Spain; Fundació Institut d'Investigació en Ciències de la Salut Germans Trias i Pujol - Universitat Autònoma de Barcelona - CIBERES, Spain

## Abstract

Cells from lung and other tissues are subjected to forces of opposing directions that are largely transmitted through integrin-mediated adhesions. How cells respond to force bidirectionality remains ill defined. To address this question, we nanofabricated flat-ended cylindrical Atomic Force Microscopy (AFM) tips with ∼1 µm^2^ cross-section area. Tips were uncoated or coated with either integrin-specific (RGD) or non-specific (RGE/BSA) molecules, brought into contact with lung epithelial cells or fibroblasts for 30 s to form focal adhesion precursors, and used to probe cell resistance to deformation in compression and extension. We found that cell resistance to compression was globally higher than to extension regardless of the tip coating. In contrast, both tip-cell adhesion strength and resistance to compression and extension were the highest when probed at integrin-specific adhesions. These integrin-specific mechanoresponses required an intact actin cytoskeleton, and were dependent on tyrosine phosphatases and Ca^2+^ signaling. Cell asymmetric mechanoresponse to compression and extension remained after 5 minutes of tip-cell adhesion, revealing that asymmetric resistance to force directionality is an intrinsic property of lung cells, as in most soft tissues. Our findings provide new insights on how lung cells probe the mechanochemical properties of the microenvironment, an important process for migration, repair and tissue homeostasis.

## Introduction

Tissue cells are subjected to a variety of dynamic mechanical stimuli during physiological processes including development, normal organ function, and in a long list of diseased conditions [1], [2]. The presence of dynamic mechanical stimuli is particularly obvious in the lung, where cells from both the parenchymal and the stromal compartments continuously experience cyclic stretching forces due to breathing [1], [3]. At the cellular level, a major effect of dynamic stretch is that cells experience forces of opposing directions (i.e. bidirectional), such as compression and extension. The normal cell mechanical response to these bidirectional forces is essential for normal lung function. Conversely, a hallmark of prevalent respiratory diseases including asthma and fibrosis is an abnormal mechanical behavior of lung cells, concomitant with an impairment of lung functions [1], [3]. Rather than acting globally on tissue cells, mechanical forces are ultimately conveyed to cells locally through their adhesion sites to neighboring cells or to their surrounding extracellular matrix (ECM) [4], [5], [6]. Previous studies have highlighted the prominent role of the integrin family of transmembrane ECM receptors in conveying extracellular forces in a variety of cell types and tissues, and in orchestrating biological responses to these forces [5], [6]. Nonetheless, our current understanding of how lung cells sense and respond to bidirectional forces is still very limited [1], [7], due in part to the lack of suitable techniques to apply such forces to cells and to probe the corresponding cell mechanoresponses.

There are several approaches to study local cell-ECM mechanical interactions either at the dorsal or ventral surface of adherent cells. These approaches have provided valuable insights into the complex nature of cell-ECM mechanical interactions. However, these approaches are subjected to limitations that undermine their application to study cell mechanoresponse to force directionality, including the inability to either apply both compression and extension to cells, to provide probe-independent cell mechanical responses due to undefined probe-cell contact geometry, or both [8], [9], [10], [11], [12], [13]. A promising approach that may overcome these technical limitations is based on AFM provided with unconventional flat-ended cylindrical AFM tips (referred to as FE-AFM tips thereafter) [14]. Unlike standard sharp pyramidal AFM tips, FE-AFM tips provide well-defined contact geometry with the cell surface that remains constant and independent of the loading force. Moreover, contact elastic models for flat tips predict a linear relationship between loading force and resulting sample deformation that holds in both compression and extension, thereby facilitating data analysis [14], [15]. In addition, FE-AFM tips provide unique versatility since the tip diameter, the characteristics of the exogenous force –including directionality, time-dependence and amplitude–, and the biochemical nature of the tip surface coating can be controlled independently. However, the application of FE-AFM tips to study cell-ECM mechanical interactions is still very scarce.

The goal of this work was to study how lung parenchymal and stromal cells respond mechanically to local bidirectional forces of compression and extension by nanofabricated FE-AFM cylindrical tips. To apply mechanical stimuli to integrins specifically, tips were coated with a synthetic peptide containing the tri-aminoacid Arg-Gly-Asp (RGD) sequence, which is an integrin-specific binding domain present in fibronectin and other ECM components [16] widely used in studies of cell-ECM mechanical interactions [10], [17]. Bare tips or tips coated with non-integrin specific molecules were used as negative controls. To mimic key geometrical aspects of physiological cell-ECM adhesions, tips were nanofabricated with a cross-section area of ∼1 µm^2^ and brought into contact with the cell surface for ∼30 s, thereby enabling the formation of focal adhesion (FA) precursors [7], [18]. A protocol including a bidirectional loading regime was used to apply compression and extension to the surface of single parenchymal or stromal lung cells, and to probe cell resistance to these deforming forces by measuring the Young's elastic modulus (*E*). This protocol was previously validated on synthetic positively charged polyacrylamide (PAA^+^) gels known to exhibit linear elasticity [19].

## Materials and Methods

### Cell culture and treatments

All experiments were carried out with stromal CCD-19Lu human lung fibroblasts and parenchymal A549 human alveolar epithelial cells (ATCC, Manassas, VA). Both cell lines were cultured as described elsewhere [14], [20]. Two days before the experiments, cells were plated on 25-mm diameter glass coverslips in serum-containing culture media for 24 h, and in serum-free media one day before measurements. In some experiments, cells were treated with either latrunculin-A (latA) (2 µM), nocodazole (10 µM), EGTA (5 mM), BAPTA/AM (10 µM), phenylarsine oxide (PAO) (2 µM), or aminoguanidine (AMG) (1 mM), (Calbiochem, La Jolla, CA). All treatments or the corresponding vehicle were added to the culture medium 30 min before measurements, and were present throughout the experiment.

### PAA^+^ gels

Gel samples were prepared as previously described [21] (see details in Text S1). To render the gel positively charged, the acrylamide solution contained 70% of 40%-acrylamide (Bio-Rad) and 30% of 3-acrylamidopropyl triethylammonium-acrylamide (Sigma). The concentration of the bisacrylamide crosslinker was chosen to elicit a gel stiffness comparable to lung tissue (∼10 kPa) [22].

### Nanofabrication of cylindrical FE-AFM tips by Focused Ion Beam (FIB)

**Figure 1:**
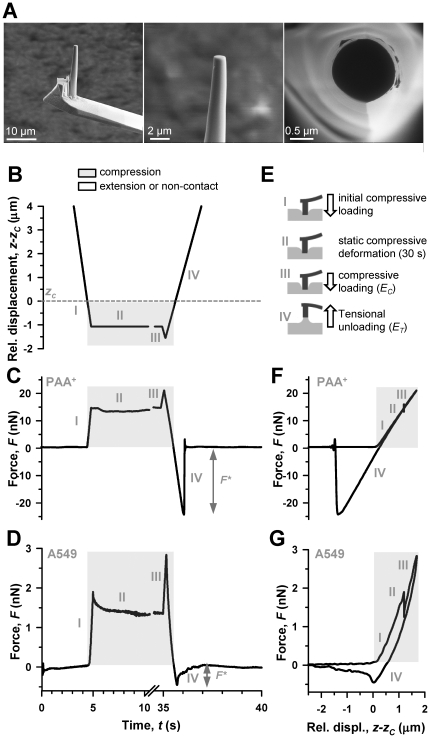
Illustration of the 4-step protocol based on FE-AFM tips used to probe cell mechanoresponses to compression and extension. (A) Representative SEM images of a nanofabricated cylindrical FE-AFM tip. A whole FIB-milled cylindrical tip is shown in the left panel, and detailed lateral and top view images of the tip are shown in the middle and right panels, respectively. (B) Driving signal of the piezotranslator in *z* as a function of time (*t*) used to probe the sample mechanoresponse to compression and extension. Note that the *z* axis was scaled relative to *z_c_*, which is marked with an horizontal dashed line. Although the relative *z* signal started at 13.5 µm, the *z* axis was limited to 4 µm above *z_c_* to better visualize the range corresponding to step III and IV. A break in the *t* axis was introduced for the same purpose. Corresponding *F* recordings as a function of *t* on a PAA^+^ gel and a single A549 cell are shown in (C) and (D), respectively. A common *t* axis was used in (B–D). F* was obtained from step IV as illustrated in (C, D). (E) Cartoon describing the tip-sample mechanical interactions corresponding to the 4-steps of the experimental protocol. *E_C_* and *E_T_* were calculated using signals from step III and IV. *F* signals from (C) and (D) were plotted against *z* in (F) and (G), respectively. The parts of the *z* and *F* signals obtained in compression were highlighted in gray. All *F* data were scaled relative to the corresponding zero force (*k⋅d_0_*).

The pyramidal tip of silicon cantilevers (MikroMasch, Tallinn, Estonia) was shaped into flat-ended cylinders by FIB following a slightly modified two-step procedure reported elsewhere [14]. First, a cylinder was obtained by milling the tip applying a ring-like ion beam (1 µm inner radius, 2.5 µm outer radius) centered on the tip apex with a tilt angle of 11°, dwell time of 10 s, and beam intensity of ∼3000 pA. This step was repeated by reducing progressively the outer diameter. Second, a flat ended tip was obtained by milling a straight line pattern (∼1000 pA) perpendicular to the cylinder axis. Finally, Scanning Electron Microscopy (SEM) images of the tips (Figure 1A) were acquired to assess the actual radius, and to measure the height and circularity of the cross-section with SEM software navigation tools.

### Modification of FE-AFM tips

For measurements with PAA^+^ gels, tips were rendered negatively charged by cleaning with piranha solution (30 min), rinsing in distilled water, and cleaning with low power oxygen plasma in a plasma cleaner-sterilizer (PDC-002, Harrick Scientific Corporation, Ossining, NY) for 1 min. For peptide or protein coatings, tips were first cleaned using piranha solution (30 min), acetone (5 min) and UV irradiation (15 min). Second, the cantilevers were soaked in a solution of 5% 3-aminopropyltriethoxysilane (Fluka, Buchs, Switzerland) in acetone, rinsed with distilled water and shaken for 30 min in a solution of 0.5% glutaraldehyde (Sigma), washed with distilled water and air dried. Finally, the cantilevers were immersed overnight at 4°C in 0.1 mg/ml solutions containing either RGD (GRGDSP-peptide, Calbiochem, Giessen, Germany), an RGD mutant containing the tri aminoacid Arg-Gly-Glu (RGE) (GRGES-peptide, Peptides International, Louisville, KY) or bovine serum albumin (BSA) (Sigma).

### AFM setup

The details of AFM setup are given elsewhere [14]. In brief, the setup was provided with a long range linearized piezoelectric translator to control the tip position with respect to the sample. The deflection (*d*) of the cantilever was measured with the optical beam deflection method. A control code written in LABVIEW (National Instruments, Austin, TX) was used to generate the driving signal for the piezoelectric position and to acquire both the actual piezoelectric vertical displacement (*z*) and *d* signals. For each cantilever, the actual spring constant (*k*) was calibrated using the thermal fluctuations method [14], and used to calculate the force (*F*) as *F = kd*.

### AFM measurements

For each cell, we first recorded three standard force-displacement (*F-z*) curves on the perinuclear region of the cell surface (Figure S1), and used these curves to assess the tip-sample contact point (*z_c_*) and the deflection offset at zero-force (*d_0_*) as previously described [23]. Second, the piezotranslator was lifted 13.5 µm above *z_c_*, and *F* was recorded as a function of time (*t*) while this four-step protocol was applied (Figure 1B, E): (step I) the tip was brought back in contact with the cell by moving the piezotranslator forward with a 14.5 µm amplitude ramp at 3 µm/s, eliciting a maximum indentation (*δ*)≤1 µm; (step II) the piezotranslator position was held for 30 s to enable the formation of FA precursors; (step III) the piezotranslator was moved forward further with a 0.5 µm amplitude ramp at 3 µm/s, and (step IV) backward at the same speed with a 15 µm amplitude ramp. The data collected during step III and IV were used to assess *E* in compression (*E_C_*) and in extension (*E_T_*). In these conditions, the hydrodynamic viscous drag force on the cantilever was negligible [24]. For each tip surface modification, AFM measurements were repeated on at least two independent cell samples (n = 12). The same protocol was applied on PAA^+^ gels. In some experiments, the four-step protocol was slightly modified to control the force during step II by manually adjusting the position of the piezotranslator with small steps to keep the position of the laser reflected off the surface of the cantilever on the photodetector within 5% of its position at the beginning of step II for 5 min (referred to as force control protocol).

### AFM data processing

The contact elastic theory for a flat-ended rigid cylinder deforming an incompressible elastic half space predicts a constant contact area (*A*) given by *A = πa* ^2^, and a *F*(*δ*) equation given by *F* = *kd_0_*+2*Eaδ*/(1−*ν* ^2^) [14], [25], where *E* is the Young's elastic modulus of the sample, *a* is the cylindrical tip radius, and *ν* is the sample's Poisson ratio (assumed to be 0.5). The *F*(*δ*) equation applies to both compression and extension, and was used to analyze all AFM measurements. Sample deformation (referred to as indentation in compression) was computed as *δ = z−z_c_−*(*d−d_0_*). Time recordings exhibiting sudden (<1 s) force variations (>0.1 nN) in step II were discarded, since these behaviors depart from the assumptions of the model. Discarded recordings were ∼30% for cells and 0% for PAA^+^ gels. *E_C_* was calculated by fitting the *F*(*δ*) equation to (*F,δ*) data in step III corresponding to a range in *z* of the loading ramp defined by 1–1.5 µm below *z_c_* (Figure 1F, G). *E_T_* was calculated by fitting the *F*(*δ*) equation to (*F,δ*) data in step IV corresponding to the range in *z* of the unloading ramp between 0.2 µm above *z_c_* and *z* of the first detectable tip-sample detachment. For gel measurements, *E_T_* was assessed using the range in *z* defined by 1–1.5 µm above *z_c_*. The different *z* range used to assess *E_T_* in cells and in gels was due to the fact that *z* corresponding to the first tip-sample detachment was smaller than 0.7 µm above *z_c_* in cells, whereas it was larger than 1.5 µm in PAA^+^ gels. To assess the asymmetry between *E_C_* and *E_T_*, we computed the ratio *E_C_*/*E_T_*. *F* data recorded during step II were analyzed in terms of stress relaxation by fitting a power-law *F*∼*t^−α^* to the last 20 s to avoid any transient artifact. The tip-sample detachment force (F*) (Figure 1C, D) was computed as previously described [14]. The linearity of *E* in compression within the *z* range covered in step III (0.5–1.5 µm) was confirmed in both cells and gels as reported elsewhere [14] (Figure S2).

### Statistics

Paired comparisons between *E_C_* and *E_T_* were carried out using paired Student's t-test. For a given loading condition, the effect of peptide or protein coating versus bare was assessed by unpaired Student's *t*-test. Statistical analyses were performed with SigmaStat software (Systat Software, Richmond, CA). Statistical significance was assumed at *p*<0.05, where *p* is the probability of the null hypothesis [26]. Unless otherwise stated, data are given as mean ± SE.

## Results

### Nanofabrication of flat-ended cylindrical tips by FIB

Our nanofabrication protocol yielded highly reproducible FE-AFM tips, with an average radius *a* = 0.53±0.26 µm, circularity *C* = 0.86±0.05, and height *h* = 11±3 µm (mean ± SD). SEM images of a representative FE-AFM tip at different magnifications are shown in Figure 1A. The smooth edge and flatness of the tip ensured that the tip-sample contact area remained constant throughout the measurements both in compression and extension.

### PAA^+^ gel stiffness is independent of the directionality of the applied force

**Figure 2:**
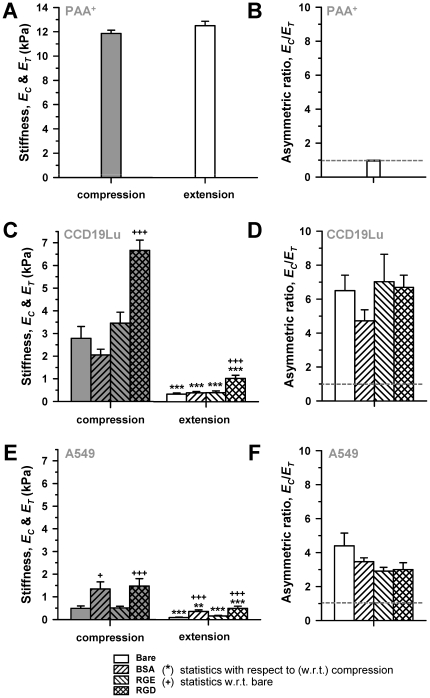
Local cell stiffness depends on the directionality and integrin-specificity of the applied force in lung cells but not in PAA^2+^ gels. (A) Average resistance to compression (*E_C_*) and extension (*E_T_*) obtained in PAA^+^ gels with negatively-charged FE-AFM tips. (B) Corresponding *E_C_*/*E_T_* data. The dashed line indicates the perfectly symmetric mechanoresponse (*E_C_* = *E_T_*). (C, E) *E_C_* and *E_T_* data probed with either bare or coated FE-AFM tips on (C) CCD-19Lu fibroblasts or (E) A549 parenchymal cells. (D, F) *E_C_*/*E_T_* data corresponding to (C) and (E), respectively. The legend of the bar filling patterns shown at the bottom apply to plots A–F. *P<0.05, **P<0.01 and ***P<0.005 were determined by paired Student's *t*-test with respect to (w.r.t.) compression. +*P*<0.05, ++*P*<0.01 and +++*P*<0.005 were determined by unpaired Student's *t*-test w.r.t. bare.

The 4-step protocol based on FE-AFM tips was first validated on PAA^+^ gels with *E_C_* comparable to human lung tissue. A representative time-recording of *F* on a PAA^+^ gel measured with a negatively charged FE-AFM tip driven in *z* according to Figure 1B is shown in Figure 1C. The sudden changes observed in *F* at ∼5 s and ∼35 s correspond to the short-term compressive loadings applied after the first contact or after holding *z* position for 30 s, respectively. Due to unspecific electrostatic interactions between the charged surfaces of the tip and the PAA^+^ gel, a net attractive force was developed between them, resulting in large adhesion forces as the F*∼−20 nN displayed in the unloading ramp beyond *z_c_* in Figure 1C. This large adhesion enabled calculating *E_T_* around an operating point (∼1.5 µm above *z_c_*) symmetric to that used to calculate *E_C_*. Figure 1F shows an *F* versus *z* plot corresponding to Figure 1B–C. The average *E* data obtained from this protocol are given in Figure 2A. These measurements clearly show several hallmarks of linear elastic materials. First, a linear relationship between *F* and *z* as predicted by the *F*(*δ*) equation (Figure 1F). Second, the overlap between the loading and unloading curves, which indicate lack of hysteresis due to viscous dissipation, (Figure 1F). Third, the absence of stress relaxation in step II (Figure 1C), indicating the time-independence of *E* at least in the time window of our experiments. Finally, we did not find any statistically significant difference among *E* measured in compression and extension (Figure 2A). Consistently, the average ratio *E_C_*/*E_T_* was equal to 1 (Figure 2B), indicating that *E* was symmetric, i.e. independent of the force directionality. Comparable results were observed using RGD-coated FE-AFM tips (Figure S3), confirming that the linear elasticity measured in PAA^+^ gels was not compromised by the coating of the FE-AFM tip.

### Local cell stiffness depends on the directionality of the applied force

Unlike PAA^+^ gels, all cell types displayed several hallmarks of viscoelastic behavior. First, *F* decayed markedly and smoothly in step II (Figure 1D), indicating that cells exhibit stress relaxation when subjected to sustained compression. Similar decays were observed with either uncoated and coated tips. Likewise, the time-dependence of the decay in *F* for all tip coatings was well fitted to a power-law with a small exponent *α*∼0.14–0.20 (r^2^≥0.9), in agreement with previous findings in A549 and other cell types [23]. The stress relaxation of the A549 cell seen in Figure 1D is displayed as a sudden drop in *F* in Figure 1G (step II); accordingly, this drop should not be mistakenly interpreted as the tip penetrating the cell. Second, Figure 1G showed hysteresis, indicating dissipation of mechanical energy due to internal viscous friction. Unlike in PAA^+^ gels, tip detachment from the cell was not abrupt, but rather smooth (Figure 1G).

To examine how lung cells respond to force directionality, we compared *E_C_* and *E_T_* measured at both integrin-specific and non-specific adhesion sites. Unlike PAA^+^ gels, the mechanoresponse of all cell types to compression and extension was markedly non-symmetric (Figure 2C–F). *E_C_* in CCD-19Lu fibroblasts were six-fold larger than *E_T_* regardless the tip-coating (Figure 2D), and their difference was statistically significant in all conditions (Figure 2C). Similar results were found in A549 cells, although the ratio *E_C_*/*E_T_* was smaller than in CCD-19Lu cells (Figure 2D, F). Likewise, all *E* data were at least four-fold smaller in A549 (Figure 2E) compared to CCD-19Lu cells (Figure 2C), which is unsurprising given the well established force generating role of fibroblasts [20]. These data indicate that the mechanical asymmetric response between compression and extension is not restricted to a single cell type and is independent of the biochemical nature of the coating.

To examine whether the asymmetric resistance to force directionality observed with RGD-coated tips was due to insufficient contact time to form mature FAs, which are typically observed after few minutes in culture [6], [27], [28], we controlled the force during step II to held RGD-coated tips in contact with CCD19-Lu fibroblasts for 5 min. A representative force recording versus time during step III and IV after applying force control in step II is shown in Figure S4. Using this protocol we observed an increase in the asymmetric ratio (*E_C_*/*E_T_* = 51±17) more than five-fold compared to the average *E_C_*/*E_T_* value observed after 30 s of tip-cell adhesion (Figure 2D). This raise was due to the marked increase in *E_C_* (19±6 kPa), whereas *E_T_* (0.53±0.04 kPa) and the adhesion strength force F* (0.79±0.04 nN) exhibited little or no alterations compared to their values after 30 s of tip-cell contact. These results suggest that, unlike *E_C_*, both *E_T_* and F* reached plateau values after ∼30 s of contact time.

### Local cell stiffness depends on the integrin-specificity of the applied force

**Figure 3:**
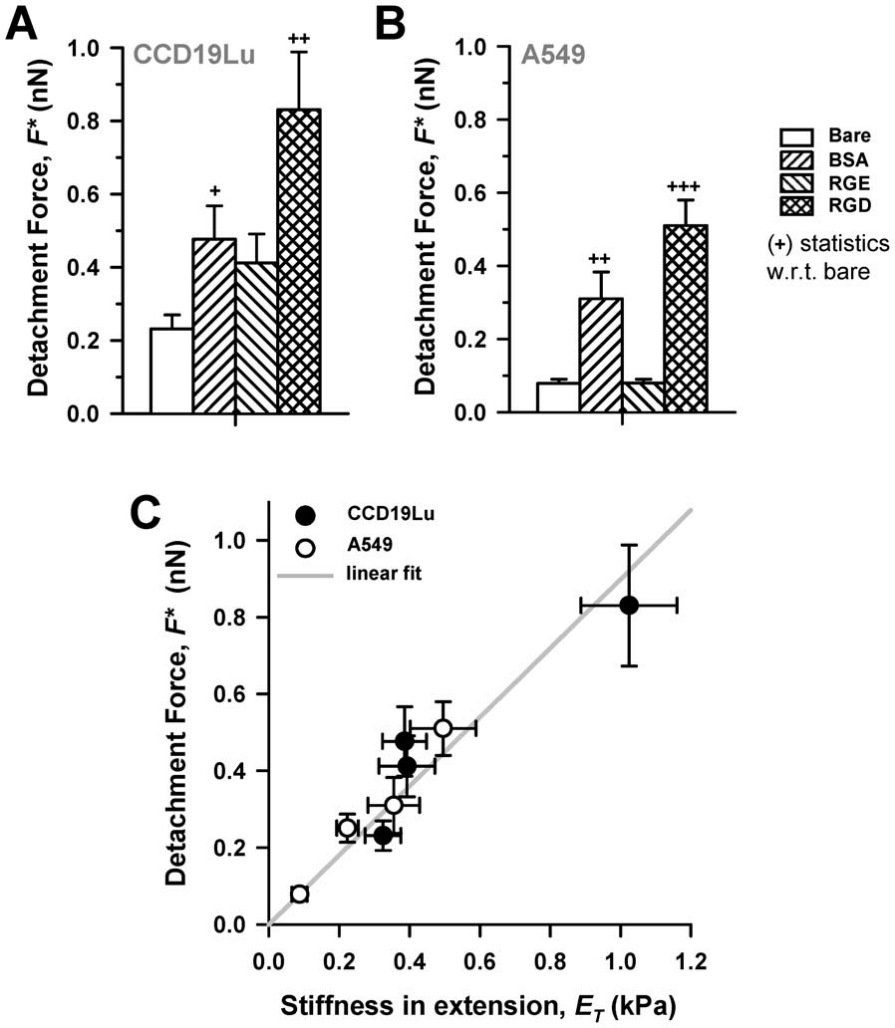
RGD-induced strengthening of lung cell-tip adhesion. (A, B) F* data obtained on (A) CCD-19Lu fibroblasts and on (B) A549 cells. Bar filling patterns shown on the right apply to plots A–B. (C) Plot of F* vs *E_T_* average data collected on all cell types with all tip coatings. Solid line corresponds to a least-squares fitting of a linear polynomial to the data. +*P*<0.05, ++*P*<0.01 and +++*P*<0.005 were determined by unpaired Student's *t*-test w.r.t. bare.

A common feature observed in both compression and extension measurements was that RGD-coated tips elicited *E* values that were two-fold larger than bare or RGE-coated tips in all cell types (Figure 2C, E), indicating that this integrin-specific stiffening was not restricted to a single cell type. Likewise, F* was at least two-fold higher when probed with RGD- than with bare or RGE-coated tips (Figure 3A, B). Plotting F* against *E_T_* from all conditions and cell types (Figure 3C) revealed that the positive correlation between *E_T_* and F* was not specific for RGD-coated tips. This correlation was well captured by fitting a linear function (F* = 0.899⋅*E_T_*, r^2^ = 0.89), as illustrated by the solid line in Figure 3C. Unexpectedly, BSA-coating, elicited a raise in *E_T_* and F* comparable to RGD in A549 cells only, although their absolute values were slightly smaller than those obtained with RGD (Figure 2E, Figure 3B). Likewise, BSA induced a raise in *E_T_* in these cells, although this raise was not as statistically robust as that observed with RGD.

### Role of cytoskeleton (CSK) integrity, and Ca^2+^ and tyrosine phosphorylation-dependent signaling in lung cell mechanoresponses

**Figure 4:**
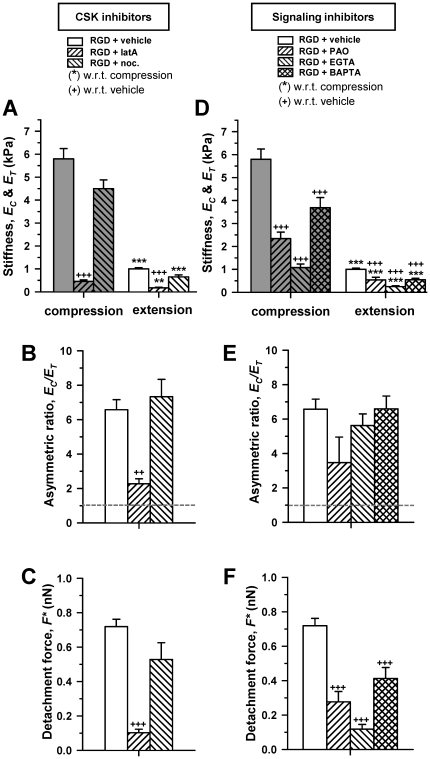
Role of cytoskeleton integrity, and Ca^2+^ and tyrosine phosphorylation-dependent signaling in lung cell mechanoresponses. (A–C) Effect of inhibitors against actin (latA) or microtubule (nocodazole) polymerization on (A) *E_C_* and *E_T_*, (B) *E_C_*/*E_T_* and (C) F* on CCD-19Lu fibroblasts probed with RGD-coated FE-AFM tips. (D-F) Effect of the tyrosine phosphatases inhibitor (PAO), and the extracellular (EGTA) and intracellular (BAPTA/AM) Ca^2+^ chelators on (D) *E_C_* and *E_T_*, (E) *E_C_*/*E_T_* and (F) F* probed in the same conditions as in (A–C). The legend of the bar filling patterns shown at the top of (A) and (D) apply to plots (A–C) and (D–F), respectively. Statistical analysis was performed as in Figure 3.

Given the prominent role of the CSK in the mechanical properties of cells [29], we examined RGD-induced cell mechanoresponses upon modification of essential cytoskeletal components with selective drugs. CCD-19Lu fibroblasts were treated with either the actin polymerization inhibitor latrunculin-A (latA), or the microtubule polymerization inhibitor nocodazole. LatA not only prevented RGD-induced stiffening in both compression and extension, but it induced a dramatic softening in all conditions, although the ratio *E_C_*/*E_T_* was dramatically reduced (Figure 4A–C). These data reveal that actin polymerization is required to elicit the largest *E_C_*, *E_T_* and F*. In contrast, compromising the integrity of the microtubules had no detectable effects in *E* or F* values probed with RGD, suggesting that an intact microtubule network was not required for driving none of the integrin-specific mechanoresponses examined here (Figure 4A–C). Similar latA-dependent features were observed in A549 cells (Figure S5), confirming that the role of actin CSK is cell type-independent. In contrast, nocodazole dramatically enhanced the RGD-specific stiffening and adhesion strengthening in A549 cells, indicating that the role of microtubules is cell type dependent. Moreover, because a large body of evidence indicates that extracellular forces trigger mechanoresponses through molecular mechanisms that are often mediated by alterations in tyrosine phosphorylation and/or Ca^2+^ signaling [2], [30], [31], we probed RGD-induced mechanoresponses in CCD-19Lu cells upon treatment with either the broad spectrum tyrosine phosphatase inhibitor PAO, or with EGTA or BAPTA/AM to chelate the extracellular and intracellular Ca^2+^, respectively. We found that all these three compounds inhibited both the integrin-specific stiffening in compression and in extension (Figure 4D), and the highest F* probed with RGD-coated tips (Figure 4F). Conversely, none of the compounds compromised the *E_C_*/*E_T_* ratio (Figure 4E). Among these inhibitors, EGTA elicited the lowest *E* and F* values, underscoring the role of extracellular Ca^2+^ in integrin-specific mechanoresponses.

## Discussion

Understanding how lung parenchymal and stromal cells respond to bidirectional forces is important for both normal physiology and diseased conditions, since many prevalent pathologies including asthma, fibrosis and acute lung injury are associated with a loss of the normal balance of forces within the lung [1], [3]. Because force transmission occurs at discrete adhesion sites rather than continuously throughout the cell surface, we studied lung cell mechanoresponse to exogenous forces applied locally at the cell surface using FE-AFM tips in conditions that enabled formation of FA precursors [7].

### Suitability of FE-AFM cylindrical tips to probe cell-ECM mechanical interactions

The identification of cell mechanoresponses to force directionality relied on the nanofabrication of cylindrical FE-AFM tips and the validation of the experimental protocol. A cylindrical geometry was chosen instead of a parallelepiped because it avoids applying unwanted high mechanical stresses to the cell surface due to sharp edges. The protocol validation was accomplished by reporting several hallmarks of linear elastic materials in PAA^+^ gels, as expected based on previous findings [19]. This validation provided also a proof of principle that FE-AFM tips can be used to quantify *E* in extension, which may be a useful tool in future studies in material sciences and cell biophysics.

FE-AFM tips also enabled quantifying the tip-sample adhesion strength. Because the cross-section of the tips was much larger than either the width of PAA^+^ filaments or the size of ECM receptors [12], [18], it is conceivable that tip-sample adhesion was mediated through multiple parallel bonds in both cells and PAA^+^ samples. Nonetheless, the shape of the unloading curves beyond *z_c_* were dramatically different in gel and cell samples (Figure 1F, G), revealing the presence of distinct physicochemical processes at the tip-sample interphase. Because PAA^+^ gels contain a single monomer type, tip-PAA^+^ gel bonds are expected to exhibit similar affinities and rupture forces. Accordingly, once the extensional force is slightly higher than the rupture force of a single bond, all bonds should detach very rapidly. Consistently, we observed a sudden decay in force during unloading in PAA^+^ gels (Figure 1F), similar to single-bond rupture experiments [32]. In contrast, the cell surface is highly heterogeneous and dynamic, and contains a non-homogeneous receptor distribution which typically exhibit distinct affinities and rupture forces [12], [16], [17]. Therefore, it is expected that cell-tip bonds will detach at different times, pulling forces and locations. Accordingly, we observed a progressive and nonlinear tip detachment from the cell surface (Figure 1G). These findings revealed that sudden or progressive tip-sample detachments are indicative of tip-sample adhesion mediated by either multiple homogeneous or heterogeneous parallel bonds, respectively, and highlighted the suitability of FE-AFM tips to study quantitatively different mechanical aspects of local cell-ECM interactions.

### Lung cells respond asymmetrically to local compression and extension

To our knowledge, this is the first quantitative study of local cell mechanoresponse to force directionality. Although *E_T_* probed with RGD-coated tips was higher than with bare or RGE coating, all *E_T_* data were smaller than *E_C_* in all cell types, in marked contrast with the equal *E_C_* and *E_T_* reported in PAA^+^ gels. Accordingly, *E_C_*/*E_T_*>1 in all conditions, and largely independent of the specific coating of the tip, whereas it was cell type dependent (Figure 2D, F). These findings indicate that integrin binding to RGD is not necessary to induce the asymmetric mechanoresponse to compression and extension. We also observed that the asymmetric resistance to force directionality remained and even increased after holding an RGD-coated tip in contact with the cell for 5 minutes using a force control protocol, which is a time window that enables forming mature FAs in culture [6], [27], [28]. The mechanical interpretation of *E* data obtained with the force control protocol is less straightforward than with the protocol used in all other experiments (Figure 1B,E), since measurements performed with the former protocol may be subjected to unwanted variability sources. These sources include force variability due to the effects of mechanical drift of the AFM setup on *z_c_* that are not corrected with the force control protocol, as well as the uncontrolled stress relaxations induced specifically on each cell by the strain steps applied when adjusting the piezotranslator position during the force control. Although these limitations could introduce variability in the calculation of *E*, several pieces of evidence support that the conclusions drawn from *E* data measured with the force control protocol are not compromised by these variability sources. First, these sources are likely to affect to a similar extend *E_C_* and *E_T_*, thereby cancelling each other out when computing the ratio *E_C_*/*E_T_*. Second, the slopes in the extension region of the *F* versus *t* curve recorded with the force control protocol (Figure S4), which are proportional to *E* according to the *F*(*δ*) equation, were consistently lower than the slopes observed during compression for all cells examined, thereby indicating qualitatively that *E_C_*/*E_T_*>1. Third, the observed three-fold increase in *E_C_* after 5 min compared to *E_C_* after 30 s of tip-cell contact falls within the same range of cell stiffening due to mechanical stimuli reported elsewhere. Thus, a five-fold increase in *E_C_* measured with AFM was observed in fibroblasts upon sustained mechanical stimulation by pulling during 30 min collagen-coated magnetic beads attached to the cell surface [31]. Likewise, ∼three-fold stiffening was measured in airway smooth muscle cells upon mechanical stimulation by cyclically twisting during 60 min magnetic beads bound to the cell surface [33]. Finally, unlike *E_C_*, we observed little variations in *E_T_* after 5 min of tip-cell adhesion compared to 30 s, in agreement with the weak (∼1–1.5 fold) increase in traction force observed in airway smooth muscle cells subjected to either intermittent or transient stretch for 5 min [2].

Our force control measurements confirmed that the elastic asymmetry observed after 30 s of tip-cell contact was not due to insufficient contact time, and strongly suggests that asymmetric resistance to compression and extension may be an intrinsic property of cells, at least in the context of the lung. In support to this interpretation, a previous study reported examples of force versus relative length curves measured on a single fibroblast sandwiched between two microplates for several minutes and subjected to low frequency cycles of either compression or extension [11]. Although the effect of force directionality was not analyzed in that study, qualitative reexamination of their cyclic measurements suggests that the fibroblast exhibited smaller apparent stiffness in extension than in compression, in agreement with our observations. Furthermore, it is worth noting that the asymmetric resistance to force directionality observed in lung cells is not physiologically surprising, since asymmetric elasticity is a hallmark of a variety of non-homogenous inert materials as well as biological materials such as bones, ligaments and soft tissues [34], [35].

Further comparisons between *E_C_* and *E_T_* data revealed that there was a consistent positive correlation between them, which was linear for nearly all experimental conditions as indicated by the constant asymmetric ratio *E_C_*/*E_T_* for each cell type. In agreement with these findings, previous studies reported a positive linear correlation between traction forces and cell stiffness in human airway smooth muscle cells [2], [36] and in human lung endothelial cells [37]. However, we found that this linearity is not universal, since *E_C_*/*E_T_* was dramatically reduced in all cell types specifically upon inhibition of actin polymerization (Figure 4 and Figure S5).

### Role of actin in lung cell asymmetric resistance to compression and extension

A straightforward mechanism underlying cell asymmetric resistance to compression and extension is that, as observed in tissues, different structures are involved in cell mechanoresponse to force directionality. This interpretation is further supported by the observation that *E_C_* was ∼10-fold smaller upon treatment with the actin polymerization inhibitor latA compared to vehicle, whereas *E_T_* was only ∼4-fold smaller in both CCD19-Lu and A549 cells (Figure 4 and Figure S5). These data confirm previous observations that an intact actin CSK is required to resist both compression [29], [37], [38] as well as extension applied at ECM adhesion sites [2], [5], [17]. In addition, these findings provide new insights in the role of actin in cell asymmetric mechanoresponse. First, they highlight a prominent role of actin polymerization in response to compressive loading. Second, they reveal that different processes underlie the assembly of the actin networks involved in resisting compression and extension. These latter observations were further expanded by our finding that both *E_T_* and F* reached a plateau after ∼30 s of tip-cell contact, whereas *E_C_* continued rising after 5 min of tip-cell compressive contact, in agreement with previous studies showing actin recruitment in response to an exogenous force [31]. Finally, these results indicate that the raise in *E_C_* after 5 min of tip-cell contact did not contribute to *E_T_*, thereby suggesting that the actin network assembled to resist compression is, at least in part, distinct from the actin network assembled to resist extension and provide adhesion strength.

### Integrin-specific mechanoresponses of lung cells to compression and extension

Local attachment to the ECM in cultured cells begins with a dot-like initial adhesion that progressively matures into ∼1 µm^2^ focal complex rich in integrins, actin, vinculin and few other FA proteins in a timescale of few seconds, and eventually forms larger and more tightly bound FAs [7], [27], [28], [39], [40]. Likewise, recent simulation studies of integrin dynamics on a rigid ECM substrate reported that 35 s was enough time to induce integrin bond formation and clustering close to maximum values [18]. Accordingly, we held an RGD-coated FE-AFM tip of ∼1 µm^2^ of cross-section area in contact with the cell surface for 30 s prior to examining its mechanoresponse to compression and extension. In these conditions, we expected cell mechanoresponses to be largely orchestrated by integrins in the context of FA precursors. To check the integrin-specificity of the RGD-induced mechanoresponses further, experiments were repeated with either bare tips or tips coated with non-integrin specific molecules (RGE/BSA). Using this approach, we identified two mechanoresponses with RGD-coated tips only regardless the cell type: (i) the largest resistance to compression and extension (Figure 2), and (ii) the highest cell-tip adhesion (Figure 3). Both mechanoresponses were inhibited in the presence of EGTA (Figure 4), which is consistent with the Ca^2+^ dependency of integrin adhesion [41]. On the other hand, the efficiency of integrins in probing ECM rigidity has been largely associated with their ability to physically link ECM to the actin CSK through adaptor proteins that are typically accumulated at FA precursors. We provided three pieces of evidence supporting this interpretation. First, integrin-specific mechanoresponses required an intact actin CSK, as revealed by latA experiments (Figure 4). Second, F* measured with RGD-coated tips was comparable to previous data including detachment forces reported using RGD coated microbeads on fibroblasts [12], and traction forces developed at focal complexes, whereas they were smaller than traction forces measured in FAs [27]. Third, a positive correlation was found between *E_T_* and F^*^ (Figure 3C), consistent with a positive correlation between traction force and vinculin accumulation at adhesion sites reported elsewhere [27]. Conversely, compromising the contractility generated within the actin CSK increased vinculin dissociation rate in FAs [42]. These latter positive correlations data suggest that the largest F* at integrin adhesion sites may arise from direct stiffening of the integrin linkage to the actin CSK through vinculin and/or other integrin-adaptor proteins present at FA precursors. However, this stiffening could also be supported by an increase in the number of integrins linked to the CSK [10], [27]. In addition, high F* could arise from the activity of mechanosensitive ion channels physically connected to the CSK [4], [17], [31]. Whatever the detailed mechanism might be, our data reveal that FA precursors are sufficient to orchestrate rapid (∼30 s) mechanoresponses that elicit strengthening or reinforcement of lung cell adhesions.

### Mechanochemical signals underlying integrin-specific mechanoresponses

In addition to cell adhesion to RGD, integrins and actin polymerization, we identified other mechanochemical cues required to elicit integrin-specific mechanoresponses in lung cells: (i) sustained compressive loading for ∼30 s, and (ii) tyrosine phosphatases and Ca^2+^ signaling. Conversely, we demonstrated that compression without RGD adhesion was not sufficient to induce these mechanoresponses. Likewise, exogenous cytokines and other soluble factors present in serum were not required. In this context, stiffening in a timescale of minutes is well documented in a variety of cell types in response to either shear or tensile stresses applied by ECM coated microneedles or microbeads [17], [31], [33], [43], [44]. Consistently, stiffening was observed when extension was applied to lung cells with RGD-coated tips only (Figure 2C, E). This stiffening has been associated with integrin activation through extension enhanced outside-in signaling [6], [44]. However, to our knowledge, this is the first study showing directly RGD-induced stiffening in compression, therefore suggesting that compression can also modulate integrin-specific mechanoresponses. On the other hand, our inhibitor experiments were consistent with a previous requirement of phosphatases in force-dependent strengthening of integrin-CSK connections during early adhesion in fibroblasts [6]. Furthermore, these inhibitor experiments further support that extra- and intra-cellular Ca^2+^ are likely necessary to regulate integrin-specific mechanoresponses through distinct processes. Extracellular Ca^2+^ is required for integrin binding to their ligands [41], and may be needed to facilitate alterations in Ca^2+^ influx through mechanosensitive ion channels [2], [17]. In contrast, inside-out integrin activation requires alterations in intracellular Ca^2+^ and downstream signaling [45].

### Potential mechanisms underlying BSA-induced mechanoresponses in A549 cells

Because BSA is thought to absorb non-specifically to the cell surface, force probes coated with BSA are commonly used as a negative control in studies of cell-ECM mechanical interactions [5], [10]. Accordingly, it is unsurprising that BSA-coated tips elicited mechanoresponses similar to bare or RGE-coating in lung fibroblasts (Figure 2 and Figure 3). In contrast, BSA-coated tips induced stiffening in both compression and extension in A549 cells comparable to RGD-coating (Figure 2E), although cell adhesion to BSA-coated tips was smaller than to RGD but larger than bare or RGE-coated tips (Figure 3B). A straightforward explanation for these unexpected findings could be that A549 cells do have receptors for BSA. In this context, a former study showed that A549 cells express receptors for BSA modified as advanced glycation end products (AGE) [46]. AGE formation occurs when glucose or other reducing sugars react non-enzymatically with free residues of proteins and modify them by reversible glycosylation, which may further develop into irreversibly glycated proteins or AGE [46]. To examine the possibility that BSA-induced mechanoresponses were due to BSA glycosylation driven by the glucose present in the culture medium, we measured attachment of A549 cells to BSA-coated substrata in the presence of the AGE formation inhibitor AMG [47]. Likewise, we assessed *E_C_*, *E_T_* and F* in A549 cells with BSA-coated tips in the presence of AMG (Figure S6). We observed a modest reduction in cell attachment, *E_T_* and F* in the presence of AMG compared to vehicle, which did not achieve statistical significance (*p* = 0.15 for cell attachment, *p* = 0.07 for *E_T_*, and *p* = 0.06 for F*). Based on these findings, it is possible that mechanisms other than BSA glycosylation underlie BSA-dependent mechanoresponses. We can envision other mechanisms based on known properties of BSA. First, the surface charge of BSA could trigger mechanoresponses by inducing alterations in the membrane potential of A549 cells [48], [49], since changes in membrane potential can drive local alterations in actin polymerization large enough to modify cell stiffness according to a recent study [50]. Second, serum albumin is known to act as a carrier of aminoacids and other biomolecules in the blood by facilitating their binding owing to the presence of surface-charged groups and ionic/hydrophobic sites [48]. Accordingly, it is conceivable that BSA-induced mechanoresponses are due to signaling driven by endogenous extracellular stimulatory factors expressed by A549 cells and bound to BSA during the course of an AFM experiment. However, testing these or other possible mechanisms will require further investigations.

### Biological implications of lung cell mechanoresponses

We can envision functional advantages of the cell mechanoresponses observed here based on their fitness to well-established physiological processes. First, the integrin-specific stiffening and adhesion strengthening are consistent with a general need of cells to interrogate the mechanochemical properties of the insoluble local microenvironment. These integrin-specific mechanoresponses may be particularly useful in guiding cell migration driven by a gradient in ECM stiffness (i.e. durotaxis) by either facilitating ECM mechanosensing, stabilizing cell-ECM adhesive sites, or generating migratory forces [2], [6], [10]. Second, the asymmetric response to bidirectional forces demonstrates that, at least in the timescale of 30 s, force transmission through the cell is not symmetric, unlike previously suggested [13]. This asymmetric mechanoresponse could be part of a ‘built-in’ sensing mechanism for force directionality, which might provide important cues for the maintenance of tissue architecture or for driving collective cell migration during lung development and repair.

## Supporting Information

Text S1 Supplemental Methods including the protocol to prepare PAA^+^ gels, to perform qRT-PCR analysis, and the Relative binding assay.
(PDF)

Figure S1 Illustrative bright field images of a FE-AFM tip in contact with the perinuclear region of an A549 cell or a CCD-19Lu fibroblast.
(PDF)

Figure S2 Linearity of the Young's modulus (*E*) in compression in the indentation range used in the 4-step protocol.
(PDF)

Figure S3 *E* of PAA gels probed with RGD-coated FE-AFM tips in compression and extension.
(PDF)

Figure S4 Illustrative example of *F* versus *t* recorded on a CCD19-Lu fibroblast after holding an RGD-coated FE-AFM tip in contact with the cell for 5 min using a force control protocol.
(PDF)

Figure S5 Role of the CSK integrity in RGD-induced stiffening and adhesion strengthening in A549 cells probed with RGD-coated FE-AFM tips.
(PDF)

Figure S6 Mechanoresponses of A549 cells to BSA-coated FE-AFM tips.
(PDF)
