# Supplementary material for: Integrin-Specific Mechanoresponses to Compression and Extension Probed by Cylindrical Flat-Ended AFM Tips in Lung Cells
Source: PLoS One. 2012 Feb 23;7(2):e32261. doi: 10.1371/journal.pone.0032261 (PMC3285695; doi:10.1371/journal.pone.0032261)
Supplement: Text S1 — Supplemental Methods including the protocol to prepare PAA+ gels, to perform qRT-PCR analysis, and the Relative binding assay. (PDF) [file pone.0032261.s001.pdf]

## SUPPLEMENTARY MATERIALS

### $PAA^+$ gels

Gel samples were prepared as previously described (20). In brief, ~70 μm thick and 8 mm in diameter $PAA^+$ gels were prepared on glass coverslips by polymerizing a mixture containing 15% wt/vol acrylamide, 20% wt/vol 2% bisacrylamide (BioRad Laboratories, Hercules, CA), 6% wt/vol TEMED (Sigma-Aldrich, St. Louis, MO), and 2 % wt/vol 10% ammonium persulfate (BioRad). To render the gel positively charged, acrylamide solution contained 70% of 40%-acrylamide (Bio-Rad) and 30% of 3-acrylamidopropyl triethylammonium-acrylamide (Sigma). The concentration of the bisacrylamide crosslinker was chosen to elicit a gel stiffness comparable to lung tissue (~10 kPa). Gels were kept in PBS before the experiments and examined with the same media.

### qRT-PCR analysis

Cells were plated in triplicates, and the corresponding total RNA was isolated using the RNeasy Mini kit (QIAGEN, Valencia, CA). A total of 400 ng of RNA was used to synthesize cDNA using High Capacity cDNA Reverse Transcription Kit (Applied Biosystems). Real-time PCR reactions were performed on 50ng of each cDNA sample using TaqMan® Gene Expression Master Mix and TaqMan® gene-specific primer pairs and probe (Applied Biosystems) for genes encoding either the advanced glycosylation end product-specific receptor (AGER) (Hs00542592_g1) or the RNA polymerase II (POLR2A, used as a reference gene) (Hs00172187_m1). Reactions were carried out for 40 cycles (95°C for 15 s and 60°C for 1 min) in a 7300 Real Time PCR System (Applied Biosystems, Foster City, CA). Relative gene expression was assessed using the comparative Ct method as described elsewhere (1). In brief, a common threshold was defined in the exponential phase of the PCR amplification curves for each gene, and the corresponding threshold cycles (Ct) were obtained and averaged over each replicate. Relative AGER gene expression with respect to POLR2 was assessed as $2^{-\Delta Ct}$.

### Relative binding assay

To assess quantitatively the attachment of A549 cells on a BSA-coated substrata in different conditions, we used a modified version of a previously reported protocol (2). In brief, cells were cultured for each condition as triplicates on 96-well plates pre-coated with either 0.1 g/L BSA or fibronectin (Sigma) in the absence or presence of aminoguanidine in serum-free medium. Cells were incubated for 2h at 37°C and washed with 1X PBS. Relative cell binding on BSA with respect to fibronectin was calculated by assessing the average number of attached cells in each condition using the crystal violet colorimetric assay (3).

## SUPPLEMENTARY REFERENCES


1. Schmittgen, T. D., and K. J. Livak. 2008. Analyzing real-time PCR data by the comparative C(T) method. Nat Protoc 3:1101-1108.
2. Yamada, K. M., and D. W. Kennedy. 1984. Dualistic nature of adhesive protein function: fibronectin and its biologically active peptide fragments can autoinhibit fibronectin function. J Cell Biol 99:29-36.
3. Kueng, W., E. Silber, and U. Eppenberger. 1989. Quantification of cells cultured on 96-well plates. Anal Biochem 182:16-19.

4. Rico, F., P. Roca-Cusachs, R. Sunyer, R. Farre, and D. Navajas. 2007. Cell dynamic adhesion and elastic properties probed with cylindrical atomic force microscopy cantilever tips. J Mol Recognit 20:459-466.
