# Supplementary material for: Integrin-Specific Mechanoresponses to Compression and Extension Probed by Cylindrical Flat-Ended AFM Tips in Lung Cells
Source: PLoS One. 2012 Feb 23;7(2):e32261. doi: 10.1371/journal.pone.0032261 (PMC3285695; doi:10.1371/journal.pone.0032261)
Supplement: Figure S1 — Illustrative bright field images of a FE-AFM tip in contact with the perinuclear region of an A549 cell or a CCD-19Lu fibroblast. (PDF) [file pone.0032261.s002.pdf]

FIGURE S1

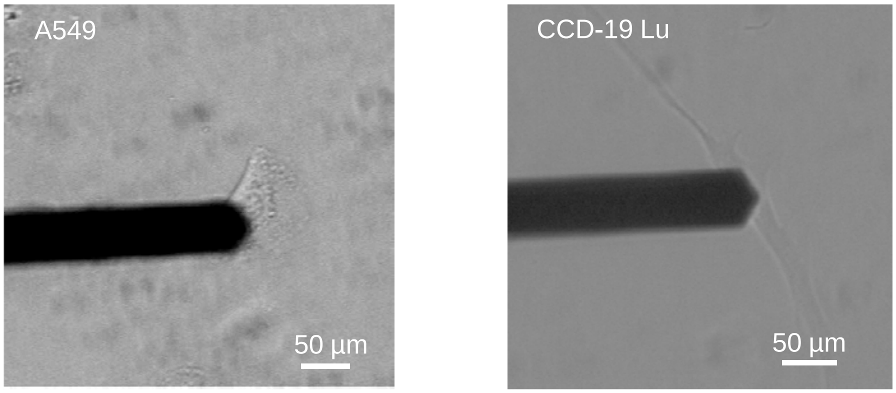

FIGURE S1. Illustrative bright field images of a FE-AFM tip in contact with the perinuclear region of an A549 cell or a CCD-19Lu fibroblast.
