# Supplementary material for: Integrin-Specific Mechanoresponses to Compression and Extension Probed by Cylindrical Flat-Ended AFM Tips in Lung Cells
Source: PLoS One. 2012 Feb 23;7(2):e32261. doi: 10.1371/journal.pone.0032261 (PMC3285695; doi:10.1371/journal.pone.0032261)
Supplement: Figure S2 — Linearity of the Young's modulus (E) in compression in the indentation range used in the 4-step protocol. (PDF) [file pone.0032261.s003.pdf]

FIGURE S2

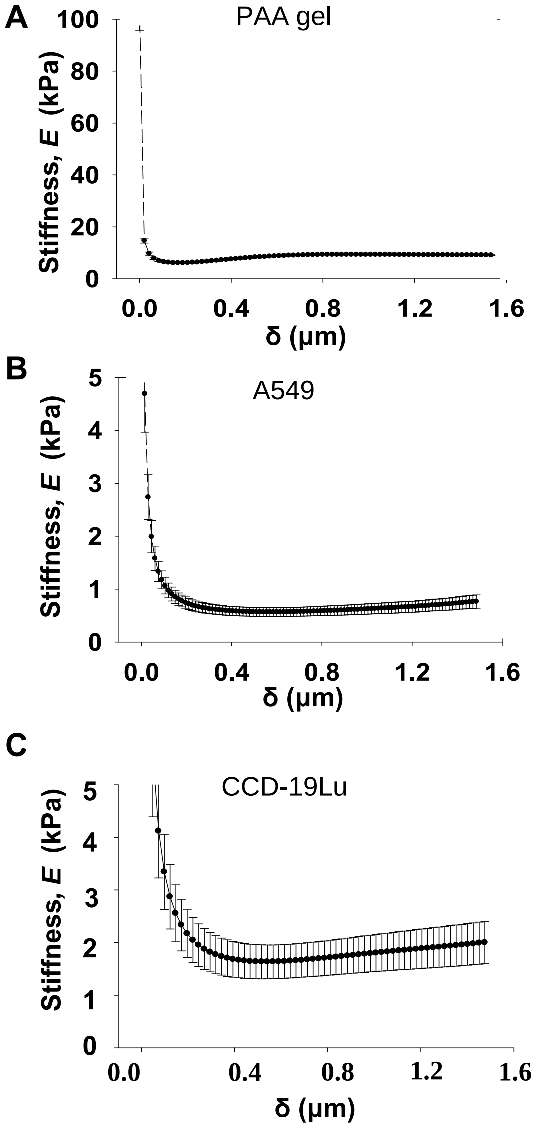

FIGURE S2. Linearity of the Young's modulus (*E*) in compression in the indentation range used in the 4-step protocol. Plot of *E* vs indentation (δ) in (A) polyacrylamide gel, (B) A549 cells, and (C) CCD-19Lu fibroblast. *E* value reach a pleateau after the initial contact point for $\delta > 0.2$ µm in gel samples, and $\delta > 0.4$ µm in both cell types, therefore including the δ range used to assess E_C.
