# Supplementary material for: Integrin-Specific Mechanoresponses to Compression and Extension Probed by Cylindrical Flat-Ended AFM Tips in Lung Cells
Source: PLoS One. 2012 Feb 23;7(2):e32261. doi: 10.1371/journal.pone.0032261 (PMC3285695; doi:10.1371/journal.pone.0032261)
Supplement: Figure S3 — E of PAA gels probed with RGD-coated FE-AFM tips in compression and extension. (PDF) [file pone.0032261.s004.pdf]

**FIGURE S3**

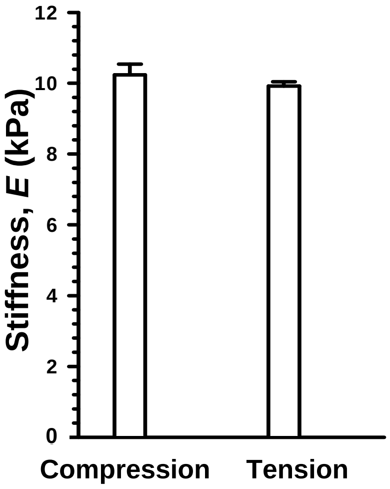


FIGURE S3. E of PAA gels probed with RGD-coated FE-AFM tips in compression and tension. *E* data were not statistically significantly different as shown by a paired Student’s t-test.
