# Supplementary material for: Integrin-Specific Mechanoresponses to Compression and Extension Probed by Cylindrical Flat-Ended AFM Tips in Lung Cells
Source: PLoS One. 2012 Feb 23;7(2):e32261. doi: 10.1371/journal.pone.0032261 (PMC3285695; doi:10.1371/journal.pone.0032261)
Supplement: Figure S4 — Illustrative example of F versus t recorded on a CCD19-Lu fibroblast after holding an RGD-coated FE-AFM tip in contact with the cell for 5 min using a force control protocol. (PDF) [file pone.0032261.s005.pdf]

FIGURE S4

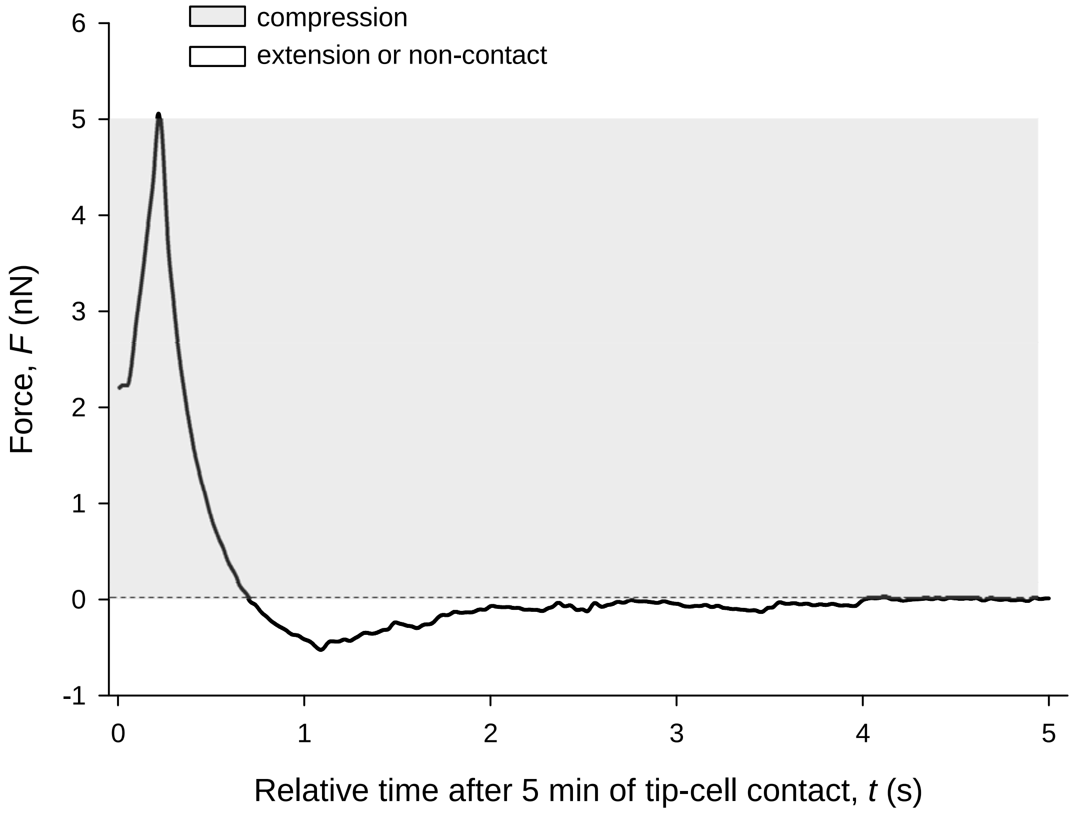

FIGURE S4. Illustrative example of force *F* versus time (*t*) recorded on a CCD19-Lu fibroblast after holding an RGD-coated FE-AFM tip in contact with the cell for 5 min using a force control protocol.
