# Supplementary material for: Integrin-Specific Mechanoresponses to Compression and Extension Probed by Cylindrical Flat-Ended AFM Tips in Lung Cells
Source: PLoS One. 2012 Feb 23;7(2):e32261. doi: 10.1371/journal.pone.0032261 (PMC3285695; doi:10.1371/journal.pone.0032261)
Supplement: Figure S5 — Role of the CSK integrity in RGD-induced stiffening and adhesion strengthening in A549 cells probed with RGD-coated FE-AFM tips. (PDF) [file pone.0032261.s006.pdf]

FIGURE S5

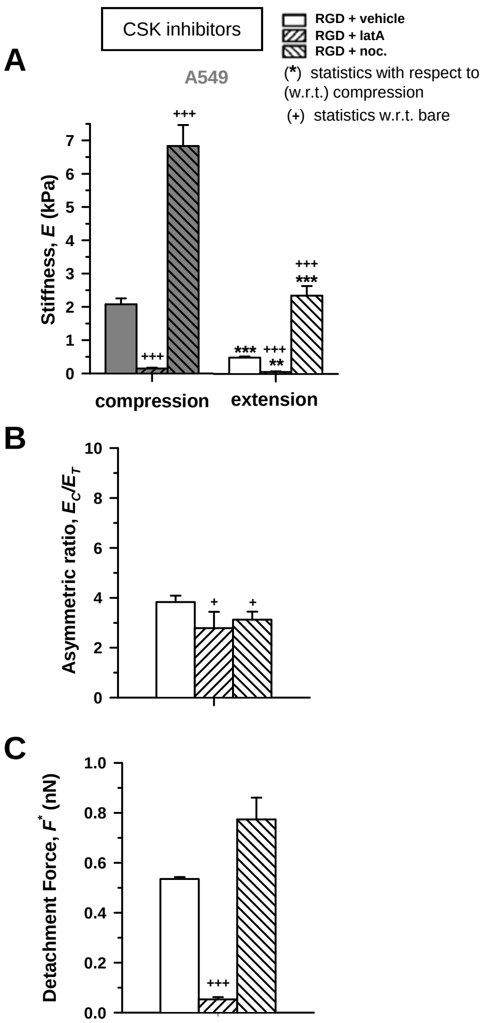

FIGURE S5. Role of the CSK integrity in RGD-induced stiffening and adhesion strengthening in A549 cells probed with RGD-coated FE-AFM tips. Effect of inhibitors against actin (latA) or microtubule (noc.) polymerization on (A) cell resistance to compression (E_C) and tension (E_T), (B) E_C/E_T and (C) cell-tip detachment force (F^*).
