# Supplementary material for: Integrin-Specific Mechanoresponses to Compression and Extension Probed by Cylindrical Flat-Ended AFM Tips in Lung Cells
Source: PLoS One. 2012 Feb 23;7(2):e32261. doi: 10.1371/journal.pone.0032261 (PMC3285695; doi:10.1371/journal.pone.0032261)
Supplement: Figure S6 — Mechanoresponses of A549 cells to BSA-coated FE-AFM tips. (PDF) [file pone.0032261.s007.pdf]

FIGURE S6

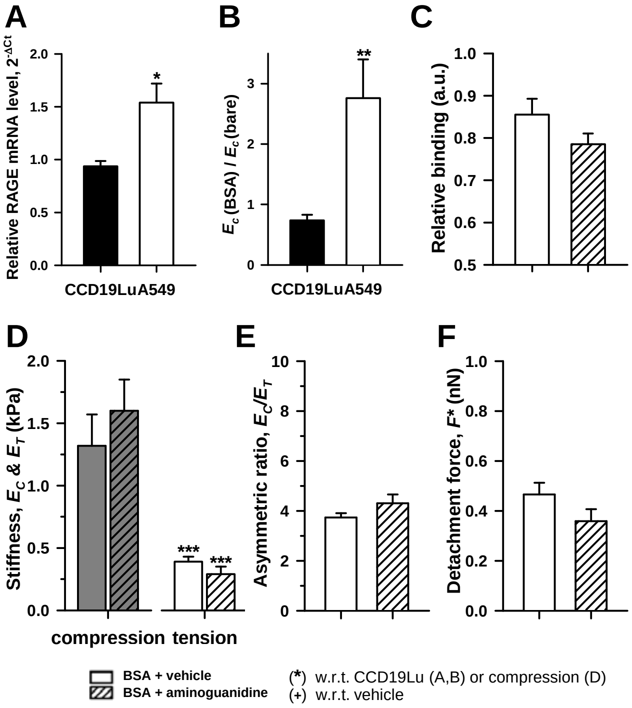

FIGURE S6. Mechanoresponses of A549 cells to BSA-coated FE-AFM tips. (A) Relative expression of RAGE in A549 cells and CCD-19Lu fibroblasts assessed by qRT-PCR as described in Suppl. Materials. (B) Normalized E_C data probed with BSA-coated tips with respect to bare tips in both cell types. (C) Relative binding of A549 cells to a BSA-coated substrata in the presence of the AGE inhibitor aminoguanidine (AMG) or vehicle, assessed as described in Suppl. Materials. Effect of AMG on (D) E_C and E_T, (E) E_C/E_T and (F) F^*. *$P < 0.05$, **$P < 0.01$ and ***$P < 0.005$ were determined by paired Student's *t*-test w.r.t. compression. +$P < 0.05$, ++$P < 0.01$ and +++$P < 0.005$ were determined by unpaired Student's *t*-test w.r.t. vehicle.
